# Tetra­kis(2-methyl­benzimidazolium) β-octa­molybdate(VI)

**DOI:** 10.1107/S1600536810012304

**Published:** 2010-04-10

**Authors:** Lujiang Hao, Xiaofei Zhang, Jiangkui Chen

**Affiliations:** a College of Food and Biological Engineering, Shandong Institute of Light Industry, Jinan 250353, People’s Republic of China

## Abstract

The asymmetric unit of the title compound, (C_8_H_9_N_2_)_4_[Mo_8_O_26_], consists of two 2-methyl­benzimidazolium cations and one-half of a *β*-Mo_8_O_26_^4−^ anion, which is completed by crystallographic inversion symmetry. An extensive net of N—H⋯O hydrogen bonds between the cations and anions contribute to the crystal packing.

## Related literature

For general background to polyoxometalates, see: Pope & Müller (1991 ▶). For polyoxometalates modified with amines, see: Zhang, Dou *et al.* (2009 ▶); Zhang, Wei *et al.* (2009 ▶). For the structures of other polyoxidomolybdates with the *β*-[Mo_8_O_26_]^4−^ anion, see, for example: Chen *et al.* (2004 ▶); Isobe *et al.* (1978 ▶); Li *et al.* (2004 ▶); Lu *et al.* (2000 ▶).
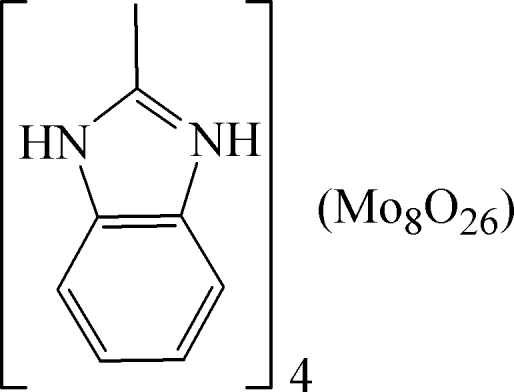


## Experimental

### 

#### Crystal data

(C_8_H_9_N_2_)_4_[Mo_8_O_26_]
*M* *_r_* = 1716.21
Monoclinic, 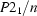

*a* = 10.4831 (12) Å
*b* = 17.803 (2) Å
*c* = 13.794 (2) Å
β = 112.305 (5)°
*V* = 2381.6 (5) Å^3^
*Z* = 2
Mo *K*α radiation
μ = 2.13 mm^−1^
*T* = 296 K
0.12 × 0.10 × 0.08 mm

#### Data collection

Bruker APEXII CCD diffractometer
Absorption correction: multi-scan (*SADABS*; Bruker, 2001 ▶) *T*_min_ = 0.784, *T*_max_ = 0.848
11151 measured reflections
4167 independent reflections
3566 reflections with *I* > 2σ(*I*)
*R*_int_ = 0.043

#### Refinement

*R*[*F* ^2^ > 2σ(*F* ^2^)] = 0.022
*wR*(*F* ^2^) = 0.063
*S* = 1.00
4167 reflections
336 parameters
H-atom parameters constrained
Δρ_max_ = 0.53 e Å^−3^
Δρ_min_ = −0.59 e Å^−3^

### 

Data collection: *APEX2* (Bruker, 2004 ▶); cell refinement: *SAINT-Plus* (Bruker, 2001 ▶); data reduction: *SAINT-Plus*; program(s) used to solve structure: *SHELXS97* (Sheldrick, 2008 ▶); program(s) used to refine structure: *SHELXL97* (Sheldrick, 2008 ▶); molecular graphics: *SHELXTL* (Sheldrick, 2008 ▶); software used to prepare material for publication: *SHELXTL*.

## Supplementary Material

Crystal structure: contains datablocks global, I. DOI: 10.1107/S1600536810012304/wm2318sup1.cif

Structure factors: contains datablocks I. DOI: 10.1107/S1600536810012304/wm2318Isup2.hkl

Additional supplementary materials:  crystallographic information; 3D view; checkCIF report

## Figures and Tables

**Table 1:** Hydrogen-bond geometry (Å, °)

*D*—H⋯*A*	*D*—H	H⋯*A*	*D*⋯*A*	*D*—H⋯*A*
N1—H1⋯O5^i^	0.86	1.95	2.811 (4)	176
N2—H2⋯O13^ii^	0.86	2.06	2.857 (4)	155
N3—H3⋯O1^i^	0.86	1.99	2.752 (4)	147
N4—H4⋯O4^iii^	0.86	2.30	3.089 (4)	154
N4—H4⋯O2^iii^	0.86	2.41	3.060 (5)	133

Symmetry codes: (i) 
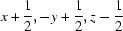
; (ii) 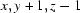; (iii) 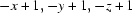.

**Fig. 1.**
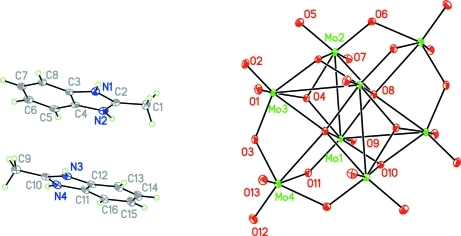
The building blocks of the title compound with the atom-labelling scheme. Displacement ellipsoids are drawn at the 30% probability level; H atoms are given as spheres of arbitrary radius.

**Fig. 2.**
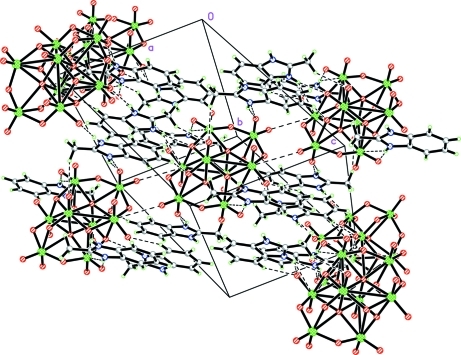
The crystal packing of the title compound, displayed with N—H···O hydrogen bonds as dashed lines.

## supplementary crystallographic information

### Comment

The design and synthesis of polyoxometalates has attracted continuous research interest not only because of their appealing structural and topological novelties, but also due to their interesting optical, electronic, magnetic, and catalytic properties, as well as their potential medical applications (Pope & Müller, 1991). In our research group, organic amines, such as 3-(2-pyridyl)pyrazole and pyrazine, are used to effectively modify polyoxomolybdates under hydrothermal condictions (Zhang, Dou *et al.*, 2009; Zhang, Wei *et al.*, 2009). Here, we describe the synthesis and structural characterization of the title compound.

As shown in Figure 1, the title compound consists of two 3-*H*-2-methylbenzimidazolium cations and one-half of a Mo_8_O_26_^4-^ anion. The octamolybdate polyanion shows a *β*-configuration with a center of symmetry. The bond lengths and angles within the anion are very similar to previously reported polyoxidomolybdates with the *β*-Mo_8_O_26_^4-^ structure (Chen *et al.*, 2004; Isobe *et al.*, 1978; Li *et al.*, 2004; Lu *et al.*, 2000). The anion can formally be bisected into two [(µ5-O)(Mo_4_O_12_)]^2-^ subunits by breaking the Mo—O^i^ bonds (-*x*, -*y*, -*z*+2). In this subunit, four Mo atoms sit approximately in a plane. There are four types of Mo—O bonds within the anion: terminal Mo—O bonds and bridging µ2-O—Mo, µ3-O—Mo, and µ5-O—Mo bonds. The corresponding bond lengths vary from the shortest with 1.686 (2) Å for one of the terminal Mo—O bonds, to the longest with 2.519 (3) Å for one of the bonds to the unusual µ5-O atom (O8) that sits in the 4 Mo plane near the center of each Mo—O moiety.

N—H···O hydrogen bonding between the cations and anions leads to a consolidation of the structure (Fig. 2; Table 1).

### Experimental

A mixture of 2-methylbenzimidazole (0.5 mmoL 0.07 g), sodium molybdate (0.4 mmoL, 0.10 g), and iron(III) chloride hexahydrate (0.25 mmol, 0.07 g) in 10 ml distilled water was sealed in a 25 ml Teflon-lined stainless steel autoclave and was kept at 433 K for three days. Colorless crystals suitable for the X-ray experiment were obtained. Anal. / calc. for C_32_H_36_Mo_8_N_8_O_26_: C, 22.37; H, 2.10; N, 6.53. Found: C, 22.10; H, 1.98; N, 6.27 %.

### Refinement

All hydrogen atoms bound to aromatic carbon atoms were refined in calculated positions using a riding model with a C—H distance of 0.93 Å and *U*_iso_ = 1.2*U*_eq_(C). Methyl H atoms were refined with a a C—H distance of 0.96 Å and 1.5*U*_eq_(C), allowing for free rotation of the methyl groups. Hydrogen atoms attached to aromatic N atoms were refined with a N—H distance of 0.86 Å and *U*_iso_ = 1.2*U*_eq_(N).

### Crystal data

**Table:**

(C_8_H_9_N_2_)_4_[Mo_8_O_26_]	*F*(000) = 1656
*M**_r_* = 1716.21	*D*_x_ = 2.393 Mg m^−^^3^
Monoclinic, *P*2_1_/*n*	Mo *K*α radiation, λ = 0.71073 Å
Hall symbol: -P 2yn	Cell parameters from 8117 reflections
*a* = 10.4831 (12) Å	θ = 2.4–30.0°
*b* = 17.803 (2) Å	µ = 2.13 mm^−^^1^
*c* = 13.794 (2) Å	*T* = 296 K
β = 112.305 (5)°	Block, colorless
*V* = 2381.6 (5) Å^3^	0.12 × 0.10 × 0.08 mm
*Z* = 2	

### Data collection

**Table:**

Bruker APEXII CCD diffractometer	4167 independent reflections
Radiation source: fine-focus sealed tube	3566 reflections with *I* > 2σ(*I*)
graphite	*R*_int_ = 0.043
φ– and ω–scans	θ_max_ = 25.0°, θ_min_ = 2.0°
Absorption correction: multi-scan (*SADABS*; Bruker, 2001)	*h* = −12→12
*T*_min_ = 0.784, *T*_max_ = 0.848	*k* = −21→16
11151 measured reflections	*l* = −16→16

### Refinement

**Table:**

Refinement on *F*^2^	Primary atom site location: structure-invariant direct methods
Least-squares matrix: full	Secondary atom site location: difference Fourier map
*R*[*F*^2^ > 2σ(*F*^2^)] = 0.022	Hydrogen site location: inferred from neighbouring sites
*wR*(*F*^2^) = 0.063	H-atom parameters constrained
*S* = 1.00	*w* = 1/[σ^2^(*F*_o_^2^) + (0.030*P*)^2^ + 3.2003*P*] where *P* = (*F*_o_^2^ + 2*F*_c_^2^)/3
4167 reflections	(Δ/σ)_max_ = 0.001
336 parameters	Δρ_max_ = 0.53 e Å^−^^3^
0 restraints	Δρ_min_ = −0.58 e Å^−^^3^

### Special details

**Table:**

Geometry. All esds (except the esd in the dihedral angle between two l.s. planes) are estimated using the full covariance matrix. The cell esds are taken into account individually in the estimation of esds in distances, angles and torsion angles; correlations between esds in cell parameters are only used when they are defined by crystal symmetry. An approximate (isotropic) treatment of cell esds is used for estimating esds involving l.s. planes.
Refinement. Refinement of *F*^2^ against ALL reflections. The weighted *R*-factor wR and goodness of fit *S* are based on *F*^2^, conventional *R*-factors *R* are based on *F*, with *F* set to zero for negative *F*^2^. The threshold expression of *F*^2^ > σ(*F*^2^) is used only for calculating *R*-factors(gt) etc. and is not relevant to the choice of reflections for refinement. *R*-factors based on *F*^2^ are statistically about twice as large as those based on *F*, and *R*- factors based on ALL data will be even larger.

### Fractional atomic coordinates and isotropic or equivalent isotropic displacement parameters (Å^2^)

**Table:**

	*x*	*y*	*z*	*U*_iso_*/*U*_eq_	
C1	0.2704 (6)	0.7102 (3)	0.2134 (4)	0.0630 (14)	
H1A	0.3083	0.7395	0.2763	0.094*	
H1B	0.2536	0.6599	0.2307	0.094*	
H1C	0.1853	0.7323	0.1677	0.094*	
C2	0.3686 (5)	0.7086 (2)	0.1602 (3)	0.0467 (11)	
C3	0.5050 (5)	0.6647 (2)	0.0825 (3)	0.0432 (11)	
C4	0.5084 (5)	0.7430 (2)	0.0804 (3)	0.0450 (11)	
C5	0.5869 (6)	0.7808 (3)	0.0347 (3)	0.0582 (14)	
H5	0.5910	0.8329	0.0340	0.070*	
C6	0.6577 (6)	0.7372 (3)	−0.0094 (4)	0.0628 (15)	
H6	0.7106	0.7608	−0.0414	0.075*	
C7	0.6544 (6)	0.6583 (3)	−0.0086 (4)	0.0577 (13)	
H7	0.7048	0.6310	−0.0394	0.069*	
C8	0.5768 (5)	0.6212 (2)	0.0378 (3)	0.0484 (11)	
H8	0.5731	0.5690	0.0388	0.058*	
C9	0.9368 (6)	0.7591 (3)	0.2339 (4)	0.0662 (15)	
H9A	0.9239	0.7804	0.1668	0.099*	
H9B	0.9127	0.7068	0.2256	0.099*	
H9C	1.0315	0.7644	0.2801	0.099*	
C10	0.8481 (6)	0.7986 (3)	0.2785 (3)	0.0553 (13)	
C11	0.7365 (5)	0.8896 (3)	0.3277 (3)	0.0504 (12)	
C12	0.7073 (5)	0.8199 (3)	0.3619 (3)	0.0535 (13)	
C13	0.6212 (6)	0.8145 (3)	0.4162 (4)	0.0681 (16)	
H13	0.5999	0.7685	0.4381	0.082*	
C14	0.5692 (6)	0.8797 (4)	0.4361 (4)	0.0759 (17)	
H14	0.5109	0.8777	0.4728	0.091*	
C15	0.5990 (6)	0.9503 (4)	0.4041 (4)	0.0716 (16)	
H15	0.5611	0.9936	0.4199	0.086*	
C16	0.6846 (6)	0.9552 (3)	0.3490 (4)	0.0592 (13)	
H16	0.7061	1.0013	0.3273	0.071*	
Mo1	0.04862 (3)	0.096451 (15)	1.00469 (2)	0.02172 (9)	
Mo2	−0.15562 (4)	0.030802 (16)	0.78252 (2)	0.02654 (9)	
Mo3	0.16768 (3)	−0.051020 (16)	0.89081 (2)	0.02505 (9)	
Mo4	0.37656 (4)	0.016654 (17)	1.11342 (3)	0.02963 (10)	
N1	0.4180 (4)	0.64678 (18)	0.1335 (3)	0.0448 (9)	
H1	0.3986	0.6018	0.1462	0.054*	
N2	0.4224 (5)	0.76737 (19)	0.1295 (3)	0.0508 (10)	
H2	0.4062	0.8136	0.1388	0.061*	
N3	0.7772 (5)	0.7662 (2)	0.3297 (3)	0.0555 (11)	
H3	0.7754	0.7188	0.3409	0.067*	
N4	0.8238 (4)	0.8718 (2)	0.2755 (3)	0.0544 (11)	
H4	0.8574	0.9042	0.2453	0.065*	
O1	0.2370 (3)	−0.13795 (14)	0.9258 (2)	0.0361 (6)	
O2	0.1516 (3)	−0.03926 (15)	0.7647 (2)	0.0384 (7)	
O3	0.3162 (3)	0.01243 (13)	0.96278 (19)	0.0307 (6)	
O4	0.0395 (3)	0.06073 (12)	0.86774 (17)	0.0251 (5)	
O5	−0.1357 (3)	0.00303 (15)	0.67023 (19)	0.0377 (7)	
O6	−0.3086 (3)	−0.02676 (13)	0.77550 (19)	0.0313 (6)	
O7	−0.2220 (3)	0.11838 (14)	0.7564 (2)	0.0405 (7)	
O8	−0.1309 (3)	0.02939 (12)	0.95578 (17)	0.0242 (5)	
O9	−0.0207 (3)	0.18273 (13)	0.97196 (19)	0.0327 (6)	
O10	0.0283 (3)	0.07826 (12)	1.13665 (17)	0.0238 (5)	
O11	0.2261 (3)	0.11265 (13)	1.05853 (19)	0.0291 (6)	
O12	0.5156 (3)	0.07420 (16)	1.1525 (2)	0.0429 (7)	
O13	0.4390 (3)	−0.07249 (15)	1.1401 (2)	0.0398 (7)	

### Atomic displacement parameters (Å^2^)

**Table:**

	*U*^11^	*U*^22^	*U*^33^	*U*^12^	*U*^13^	*U*^23^
C1	0.079 (4)	0.043 (3)	0.070 (3)	0.022 (3)	0.032 (3)	0.014 (2)
C2	0.056 (3)	0.030 (2)	0.044 (2)	0.008 (2)	0.008 (2)	0.0069 (18)
C3	0.052 (3)	0.028 (2)	0.040 (2)	−0.009 (2)	0.006 (2)	0.0075 (17)
C4	0.054 (3)	0.027 (2)	0.038 (2)	−0.007 (2)	0.001 (2)	0.0049 (17)
C5	0.071 (4)	0.038 (3)	0.050 (3)	−0.023 (3)	0.005 (3)	0.011 (2)
C6	0.070 (4)	0.057 (3)	0.055 (3)	−0.027 (3)	0.017 (3)	0.008 (2)
C7	0.059 (4)	0.057 (3)	0.053 (3)	−0.014 (3)	0.017 (3)	0.000 (2)
C8	0.060 (3)	0.035 (2)	0.048 (2)	−0.006 (2)	0.018 (2)	0.002 (2)
C9	0.078 (5)	0.060 (3)	0.057 (3)	−0.004 (3)	0.022 (3)	0.012 (3)
C10	0.056 (4)	0.055 (3)	0.042 (2)	−0.017 (3)	0.005 (2)	0.019 (2)
C11	0.037 (3)	0.054 (3)	0.049 (2)	−0.013 (2)	0.004 (2)	0.021 (2)
C12	0.045 (3)	0.059 (3)	0.045 (2)	−0.020 (3)	0.005 (2)	0.022 (2)
C13	0.065 (4)	0.071 (4)	0.067 (3)	−0.018 (3)	0.023 (3)	0.025 (3)
C14	0.058 (4)	0.103 (5)	0.073 (4)	−0.003 (4)	0.030 (3)	0.026 (4)
C15	0.057 (4)	0.078 (4)	0.075 (3)	0.007 (3)	0.020 (3)	0.023 (3)
C16	0.045 (3)	0.056 (3)	0.068 (3)	−0.003 (3)	0.012 (3)	0.023 (3)
Mo1	0.0292 (2)	0.01279 (14)	0.02839 (15)	0.00022 (12)	0.01688 (14)	0.00018 (11)
Mo2	0.0334 (2)	0.01937 (16)	0.02819 (16)	0.00220 (13)	0.01317 (15)	0.00172 (12)
Mo3	0.0308 (2)	0.01887 (15)	0.03238 (16)	0.00054 (13)	0.01981 (15)	−0.00203 (12)
Mo4	0.0279 (2)	0.02408 (17)	0.03886 (18)	0.00065 (13)	0.01489 (16)	−0.00324 (13)
N1	0.058 (3)	0.0221 (16)	0.054 (2)	0.0012 (17)	0.022 (2)	0.0097 (15)
N2	0.070 (3)	0.0210 (17)	0.049 (2)	0.0021 (18)	0.009 (2)	0.0086 (15)
N3	0.062 (3)	0.046 (2)	0.050 (2)	−0.019 (2)	0.012 (2)	0.0170 (18)
N4	0.050 (3)	0.049 (2)	0.058 (2)	−0.0124 (19)	0.014 (2)	0.0295 (19)
O1	0.0372 (18)	0.0232 (13)	0.0545 (16)	0.0030 (12)	0.0251 (14)	−0.0021 (12)
O2	0.048 (2)	0.0385 (15)	0.0393 (14)	−0.0047 (13)	0.0280 (14)	−0.0034 (12)
O3	0.0336 (17)	0.0265 (13)	0.0407 (14)	−0.0021 (11)	0.0240 (13)	−0.0025 (11)
O4	0.0326 (16)	0.0182 (11)	0.0307 (12)	−0.0016 (10)	0.0191 (12)	0.0002 (10)
O5	0.050 (2)	0.0327 (14)	0.0330 (13)	0.0010 (13)	0.0192 (13)	0.0012 (11)
O6	0.0319 (17)	0.0275 (13)	0.0342 (13)	−0.0010 (11)	0.0121 (12)	−0.0037 (11)
O7	0.044 (2)	0.0242 (14)	0.0467 (15)	0.0063 (13)	0.0105 (14)	0.0023 (12)
O8	0.0260 (15)	0.0180 (11)	0.0331 (12)	0.0023 (10)	0.0161 (11)	0.0010 (10)
O9	0.0456 (19)	0.0192 (12)	0.0389 (13)	0.0050 (12)	0.0223 (13)	0.0019 (10)
O10	0.0301 (16)	0.0175 (11)	0.0277 (11)	−0.0007 (10)	0.0154 (11)	−0.0018 (9)
O11	0.0333 (17)	0.0208 (12)	0.0385 (13)	−0.0022 (11)	0.0197 (12)	−0.0015 (10)
O12	0.0305 (18)	0.0396 (16)	0.0591 (17)	−0.0047 (13)	0.0176 (14)	−0.0087 (14)
O13	0.0398 (19)	0.0302 (14)	0.0492 (15)	0.0072 (13)	0.0167 (14)	−0.0008 (13)

### Geometric parameters (Å, °)

**Table:**

C1—C2	1.473 (7)	Mo1—O9	1.686 (2)
C1—H1A	0.9600	Mo1—O11	1.746 (3)
C1—H1B	0.9600	Mo1—O10	1.939 (2)
C1—H1C	0.9600	Mo1—O4	1.961 (2)
C2—N2	1.332 (6)	Mo1—O8	2.112 (2)
C2—N1	1.326 (5)	Mo1—O8^i^	2.388 (2)
C3—N1	1.384 (6)	Mo1—Mo3^i^	3.2178 (5)
C3—C8	1.378 (6)	Mo1—Mo2	3.2189 (6)
C3—C4	1.395 (5)	Mo2—O7	1.689 (3)
C4—C5	1.386 (7)	Mo2—O5	1.712 (2)
C4—N2	1.386 (6)	Mo2—O6	1.874 (3)
C5—C6	1.366 (8)	Mo2—O4	2.007 (3)
C5—H5	0.9300	Mo2—O8	2.304 (2)
C6—C7	1.405 (7)	Mo2—O10^i^	2.380 (2)
C6—H6	0.9300	Mo3—O2	1.696 (2)
C7—C8	1.379 (6)	Mo3—O1	1.700 (3)
C7—H7	0.9300	Mo3—O3	1.874 (3)
C8—H8	0.9300	Mo3—O10^i^	2.002 (2)
C9—C10	1.474 (8)	Mo3—O8^i^	2.322 (2)
C9—H9A	0.9600	Mo3—O4	2.354 (2)
C9—H9B	0.9600	Mo3—Mo1^i^	3.2178 (5)
C9—H9C	0.9600	Mo4—O12	1.694 (3)
C10—N4	1.326 (6)	Mo4—O13	1.703 (3)
C10—N3	1.335 (6)	Mo4—O6^i^	1.928 (2)
C11—C16	1.366 (7)	Mo4—O3	1.930 (2)
C11—N4	1.399 (6)	Mo4—O11	2.253 (3)
C11—C12	1.402 (6)	Mo4—O8^i^	2.519 (3)
C12—N3	1.377 (7)	N1—H1	0.8600
C12—C13	1.377 (7)	N2—H2	0.8600
C13—C14	1.353 (8)	N3—H3	0.8600
C13—H13	0.9300	N4—H4	0.8600
C14—C15	1.407 (8)	O6—Mo4^i^	1.928 (2)
C14—H14	0.9300	O8—Mo3^i^	2.322 (2)
C15—C16	1.381 (8)	O8—Mo1^i^	2.388 (2)
C15—H15	0.9300	O10—Mo3^i^	2.002 (2)
C16—H16	0.9300	O10—Mo2^i^	2.380 (2)
			
C2—C1—H1A	109.5	Mo3^i^—Mo1—Mo2	90.460 (15)
C2—C1—H1B	109.5	O7—Mo2—O5	104.76 (13)
H1A—C1—H1B	109.5	O7—Mo2—O6	102.64 (13)
C2—C1—H1C	109.5	O5—Mo2—O6	101.01 (12)
H1A—C1—H1C	109.5	O7—Mo2—O4	97.22 (12)
H1B—C1—H1C	109.5	O5—Mo2—O4	99.18 (12)
N2—C2—N1	107.8 (4)	O6—Mo2—O4	146.89 (10)
N2—C2—C1	127.1 (4)	O7—Mo2—O8	96.12 (11)
N1—C2—C1	125.1 (4)	O5—Mo2—O8	158.55 (11)
N1—C3—C8	132.5 (4)	O6—Mo2—O8	78.74 (9)
N1—C3—C4	105.5 (4)	O4—Mo2—O8	72.97 (8)
C8—C3—C4	122.0 (4)	O7—Mo2—O10^i^	164.81 (11)
C5—C4—N2	132.7 (4)	O5—Mo2—O10^i^	87.08 (10)
C5—C4—C3	121.2 (5)	O6—Mo2—O10^i^	83.97 (10)
N2—C4—C3	106.1 (4)	O4—Mo2—O10^i^	71.11 (9)
C6—C5—C4	116.4 (4)	O8—Mo2—O10^i^	71.52 (8)
C6—C5—H5	121.8	O7—Mo2—Mo1	86.53 (10)
C4—C5—H5	121.8	O5—Mo2—Mo1	134.48 (10)
C5—C6—C7	123.0 (5)	O6—Mo2—Mo1	119.64 (7)
C5—C6—H6	118.5	O4—Mo2—Mo1	35.30 (6)
C7—C6—H6	118.5	O8—Mo2—Mo1	40.91 (6)
C8—C7—C6	120.2 (5)	O10^i^—Mo2—Mo1	78.35 (6)
C8—C7—H7	119.9	O2—Mo3—O1	105.60 (13)
C6—C7—H7	119.9	O2—Mo3—O3	102.02 (12)
C3—C8—C7	117.2 (4)	O1—Mo3—O3	102.64 (13)
C3—C8—H8	121.4	O2—Mo3—O10^i^	98.07 (12)
C7—C8—H8	121.4	O1—Mo3—O10^i^	97.49 (12)
C10—C9—H9A	109.5	O3—Mo3—O10^i^	146.37 (9)
C10—C9—H9B	109.5	O2—Mo3—O8^i^	158.12 (11)
H9A—C9—H9B	109.5	O1—Mo3—O8^i^	95.34 (10)
C10—C9—H9C	109.5	O3—Mo3—O8^i^	78.99 (9)
H9A—C9—H9C	109.5	O10^i^—Mo3—O8^i^	72.51 (9)
H9B—C9—H9C	109.5	O2—Mo3—O4	85.84 (11)
N4—C10—N3	107.6 (5)	O1—Mo3—O4	165.57 (10)
N4—C10—C9	126.9 (4)	O3—Mo3—O4	83.04 (10)
N3—C10—C9	125.5 (5)	O10^i^—Mo3—O4	71.77 (8)
C16—C11—N4	134.1 (4)	O8^i^—Mo3—O4	72.51 (8)
C16—C11—C12	121.8 (5)	O2—Mo3—Mo1^i^	132.68 (10)
N4—C11—C12	104.1 (4)	O1—Mo3—Mo1^i^	86.67 (9)
N3—C12—C13	131.7 (5)	O3—Mo3—Mo1^i^	119.94 (7)
N3—C12—C11	107.1 (4)	O10^i^—Mo3—Mo1^i^	34.61 (6)
C13—C12—C11	121.1 (5)	O8^i^—Mo3—Mo1^i^	40.95 (6)
C14—C13—C12	116.6 (5)	O4—Mo3—Mo1^i^	79.05 (5)
C14—C13—H13	121.7	O12—Mo4—O13	106.33 (15)
C12—C13—H13	121.7	O12—Mo4—O6^i^	102.94 (12)
C13—C14—C15	123.2 (5)	O13—Mo4—O6^i^	98.20 (12)
C13—C14—H14	118.4	O12—Mo4—O3	104.89 (12)
C15—C14—H14	118.4	O13—Mo4—O3	97.98 (12)
C16—C15—C14	119.8 (6)	O6^i^—Mo4—O3	142.20 (11)
C16—C15—H15	120.1	O12—Mo4—O11	93.22 (12)
C14—C15—H15	120.1	O13—Mo4—O11	160.46 (12)
C11—C16—C15	117.4 (5)	O6^i^—Mo4—O11	76.92 (10)
C11—C16—H16	121.3	O3—Mo4—O11	76.46 (9)
C15—C16—H16	121.3	C2—N1—C3	110.6 (4)
O9—Mo1—O11	104.19 (13)	C2—N1—H1	124.7
O9—Mo1—O10	102.34 (10)	C3—N1—H1	124.7
O11—Mo1—O10	96.18 (11)	C2—N2—C4	110.0 (4)
O9—Mo1—O4	100.32 (10)	C2—N2—H2	125.0
O11—Mo1—O4	96.74 (10)	C4—N2—H2	125.0
O10—Mo1—O4	150.22 (9)	C10—N3—C12	110.0 (4)
O9—Mo1—O8	100.83 (12)	C10—N3—H3	125.0
O11—Mo1—O8	154.98 (10)	C12—N3—H3	125.0
O10—Mo1—O8	78.64 (9)	C10—N4—C11	111.2 (4)
O4—Mo1—O8	78.32 (9)	C10—N4—H4	124.4
O9—Mo1—O8^i^	175.91 (12)	C11—N4—H4	124.4
O11—Mo1—O8^i^	79.83 (10)	Mo3—O3—Mo4	117.45 (12)
O10—Mo1—O8^i^	77.76 (8)	Mo1—O4—Mo2	108.44 (11)
O4—Mo1—O8^i^	78.28 (8)	Mo1—O4—Mo3	109.11 (10)
O8—Mo1—O8^i^	75.15 (10)	Mo2—O4—Mo3	104.92 (9)
O9—Mo1—Mo3^i^	92.48 (9)	Mo2—O6—Mo4^i^	118.36 (13)
O11—Mo1—Mo3^i^	132.03 (7)	Mo1—O8—Mo2	93.48 (8)
O10—Mo1—Mo3^i^	35.91 (8)	Mo1—O8—Mo3^i^	92.93 (9)
O4—Mo1—Mo3^i^	124.42 (7)	Mo2—O8—Mo3^i^	162.00 (12)
O8—Mo1—Mo3^i^	46.11 (6)	Mo1—O8—Mo1^i^	104.85 (10)
O8^i^—Mo1—Mo3^i^	85.24 (5)	Mo2—O8—Mo1^i^	97.79 (8)
O9—Mo1—Mo2	90.71 (9)	Mo3^i^—O8—Mo1^i^	96.78 (8)
O11—Mo1—Mo2	132.94 (7)	Mo1—O10—Mo3^i^	109.48 (11)
O10—Mo1—Mo2	124.25 (7)	Mo1—O10—Mo2^i^	109.51 (9)
O4—Mo1—Mo2	36.25 (7)	Mo3^i^—O10—Mo2^i^	104.12 (9)
O8—Mo1—Mo2	45.61 (6)	Mo1—O11—Mo4	120.98 (12)
O8^i^—Mo1—Mo2	85.92 (6)		

Symmetry codes: (i) −*x*, −*y*, −*z*+2.

### Hydrogen-bond geometry (Å, °)

**Table:**

*D*—H···*A*	*D*—H	H···*A*	*D*···*A*	*D*—H···*A*
N1—H1···O5^ii^	0.86	1.95	2.811 (4)	176
N2—H2···O13^iii^	0.86	2.06	2.857 (4)	155
N3—H3···O1^ii^	0.86	1.99	2.752 (4)	147
N4—H4···O4^iv^	0.86	2.30	3.089 (4)	154
N4—H4···O2^iv^	0.86	2.41	3.060 (5)	133

Symmetry codes: (ii) *x*+1/2, −*y*+1/2, *z*−1/2; (iii) *x*, *y*+1, *z*−1; (iv) −*x*+1, −*y*+1, −*z*+1.
